# Adding Fuel to the Fire: The Impacts of Non-Native Grass Invasion on Fire Management at a Regional Scale

**DOI:** 10.1371/journal.pone.0059144

**Published:** 2013-05-14

**Authors:** Samantha A. Setterfield, Natalie A. Rossiter-Rachor, Michael M. Douglas, Lisa Wainger, Aaron M. Petty, Piers Barrow, Ian J. Shepherd, Keith B. Ferdinands

**Affiliations:** 1 Research Institute for Environment and Livelihoods, Charles Darwin University, Darwin, Northern Territory, Australia; 2 University of Maryland Center for Environmental Science, Solomons, Maryland, United States of America; 3 Weed Management Branch, Northern Territory Department of Land Resource Management, Darwin, Northern Territory, Australia; 4 Australian Bureau of Meteorology, Casuarina, Northern Territory, Australia; USDA-ARS, United States of America

## Abstract

**Background:**

Widespread invasion by non-native plants has resulted in substantial change in fire-fuel characteristics and fire-behaviour in many of the world's ecosystems, with a subsequent increase in the risk of fire damage to human life, property and the environment. Models used by fire management agencies to assess fire risk are dependent on accurate assessments of fuel characteristics but there is little evidence that they have been modified to reflect landscape-scale invasions. There is also a paucity of information documenting other changes in fire management activities that have occurred to mitigate changed fire regimes. This represents an important limitation in information for both fire and weed risk management.

**Methodology/Principal Findings:**

We undertook an aerial survey to estimate changes to landscape fuel loads in northern Australia resulting from invasion by *Andropogon gayanus* (gamba grass). Fuel load within the most densely invaded area had increased from 6 to 10 t ha^−1^ in the past two decades. Assessment of the effect of calculating the Grassland Fire Danger Index (GFDI) for the 2008 and 2009 fire seasons demonstrated that an increase from 6 to 10 t ha^−1^ resulted in an increase from five to 38 days with fire risk in the ‘severe’ category in 2008 and from 11 to 67 days in 2009. The season of severe fire weather increased by six weeks. Our assessment of the effect of increased fuel load on fire management practices showed that fire management costs in the region have increased markedly (∼9 times) in the past decade due primarily to *A. gayanus* invasion.

**Conclusions/Significance:**

This study demonstrated the high economic cost of mitigating fire impacts of an invasive grass. This study demonstrates the need to quantify direct and indirect invasion costs to assess the risk of further invasion and to appropriately fund fire and weed management strategies.

## Introduction

### Assessing the risk of non-native grass invasions in fire-prone ecosystems

Non-native grass invasions have resulted in major changes to community structure and function in many of the world's ecosystems [1]. In fire-prone environments, one of the most significant consequences occurs when the invader substantially changes fire fuel properties, subsequently changing fire behaviour and fire regimes [2]–[4]. The term ‘grass - fire cycle’ [5] was coined to describe the situation when the altered fire regime created conditions detrimental to maintenance of native species and favourable to establishment and spread of the non-native plant [6]. Various authors have described dramatic ecological consequences of this cycle but there is a lack of information on other important economic, social and cultural impacts [7], particularly when invasion extends to the landscape or regional level. Without a full understanding of the range of important impacts, managers cannot adequately assess the risk of invasion and therefore determine the appropriate level of investment to prevent further invasion or to mitigate invasion impacts such as fire management. Assessments should be undertaken from the earliest stages of invasion to inform and improve risk management [8].

### The threat of non-native grasses to Australia's savannas

Tropical savanna ecosystems are characterised by frequent burning (every 1–3 years) because profuse production of native herbaceous plants during the wet season results in large amounts of fine fuel available annually to carry fire [9]. Non-native grass invasion is considered a significant ecological threat to the world's savannas, particularly in the neotropics and Australia [10], [11] due to impacts on fire regimes. The Australian savanna is the world's largest intact area of savanna and contains sites that are internationally recognised for their biodiversity and cultural significance, such as the World Heritage listed, Kakadu National Park [12]. The invasion threat is posed by a number of high-biomass non-native grasses; five of these were recently listed as a Key Threatening Process to Australia under Federal legislation [13]. Currently, the greatest threat is the African grass *Andropogon gayanus* Kunth. (gamba grass). Spread has been rapid since the 1990s but it is considered to still be in the relatively early stages of invasion [14] with modelling predicting that most of the country's vast area of savanna is suitable for invasion, including ∼380,000 km^2^ of the Australia's Northern Territory [15], as well as large savanna areas in Queensland and Western Australia [11]. *Andropogon gayanus* invasion greatly alters the fuel bed characteristics of savanna communities, replacing the short (≈0.5 m), native grass fuel bed (up to 6 t ha ^−1^) [23], with a tall (≈4 m), dense fuel bed of up to 30 t ha^−1^ [14], [16], [18]. As a result, fire intensity (the product of the available heat of combustion per unit of ground area and the forward spread of the fire, measured in kilo or megawatts per metre) increases significantly, from typically 1–3 MW m^−1^ in native grass fires to 16 MW m^−1^ in *A. gayanus*-fuelled fires in the early dry season [16]. To date, attention has focussed on documenting the detrimental ecological impacts from site-scale comparisons of invaded and non-invaded savanna, particularly the substantial decline in the diversity and abundance of native vegetation, reduction in above-ground carbon stores and changes in nitrogen cycling [16], [17]. Changes resulting from *A. gayanus* invasion at a regional scale need to be assessed, particularly the risk of fire and the ability of fire managers to protect the community and the region's environmental, economic and cultural assets. In this study, we quantify changes to fuel load and fire risk at a regional scale, and the consequences of this change to fire management

### Managing fire risk in Australia's savannas

In the early dry season, Australia's savanna fire managers begin major programs of fuel reduction burning to reduce the risk of high intensity fires later in the dry season. Early dry season fires are typically of low severity, patchy and easily extinguished; therefore, fuel reduction burning requires few management resources. As the dry season progresses, fires typically become more intense and cover larger areas, and fire management across northern Australia shifts to primarily controlling wildfires which requires more fire-fighting personnel and specialized equipment. Wildfires cause significant social and economic impacts such as the loss of grass fodder for livestock, damage to public and private infrastructure, impact on sensitive vegetation communities (e.g. rainforest patches) and cultural sites. Late dry-season fires also release approximately double the greenhouse gas emissions of low severity fires and multi-million dollar investments are now funding strategic fire management programs by the region's Indigenous communities with the aim of reducing the area of high severity fires [19].

Fire management authorities use a fire danger index to base their assessment of fire risk and their operational response, such as fire risk warnings to the public and assessment of staffing levels of fire response crews [20]. In northern Australia, the index used is the McArthur Mark 4 Grassland Fire Danger Index (GFDI; [21], [22]) which is calculated daily by Australia's Bureau of Meteorology (BOM) based on weather conditions and characteristics of the fuel (e.g. quantity, moisture content). In the savanna region of the Northern Territory (north of 17°S), the GFDI is calculated using a standard native grass fuel load of 6 t ha^−1^; this measure was established by determining the quantity of fine fuel within a defined area [23]. Given the importance of the fuel load parameter for calculating GFDI, we undertook an intensive aerial survey to determine whether there was a detectable increase in fuel load within a region of dense *A. gayanus* invasion. Fire managers had reported that their budgets were being stretched because they were assigning more staff and fire-fighting resources to *A. gayanus*-fuelled fires. This raised the question about whether the economic consequences of changed fire behaviour were considered in the implementation and resourcing of *A. gayanus* control strategies, or whether there is a disconnect between the two. Therefore, in this study, we aimed to assess the effect of *A. gayanus* invasion on regional fuel load and the consequences of this invasion on GFDI, fire management practices and their associated costs.

## Methods

### Study System

**Figure 1:**
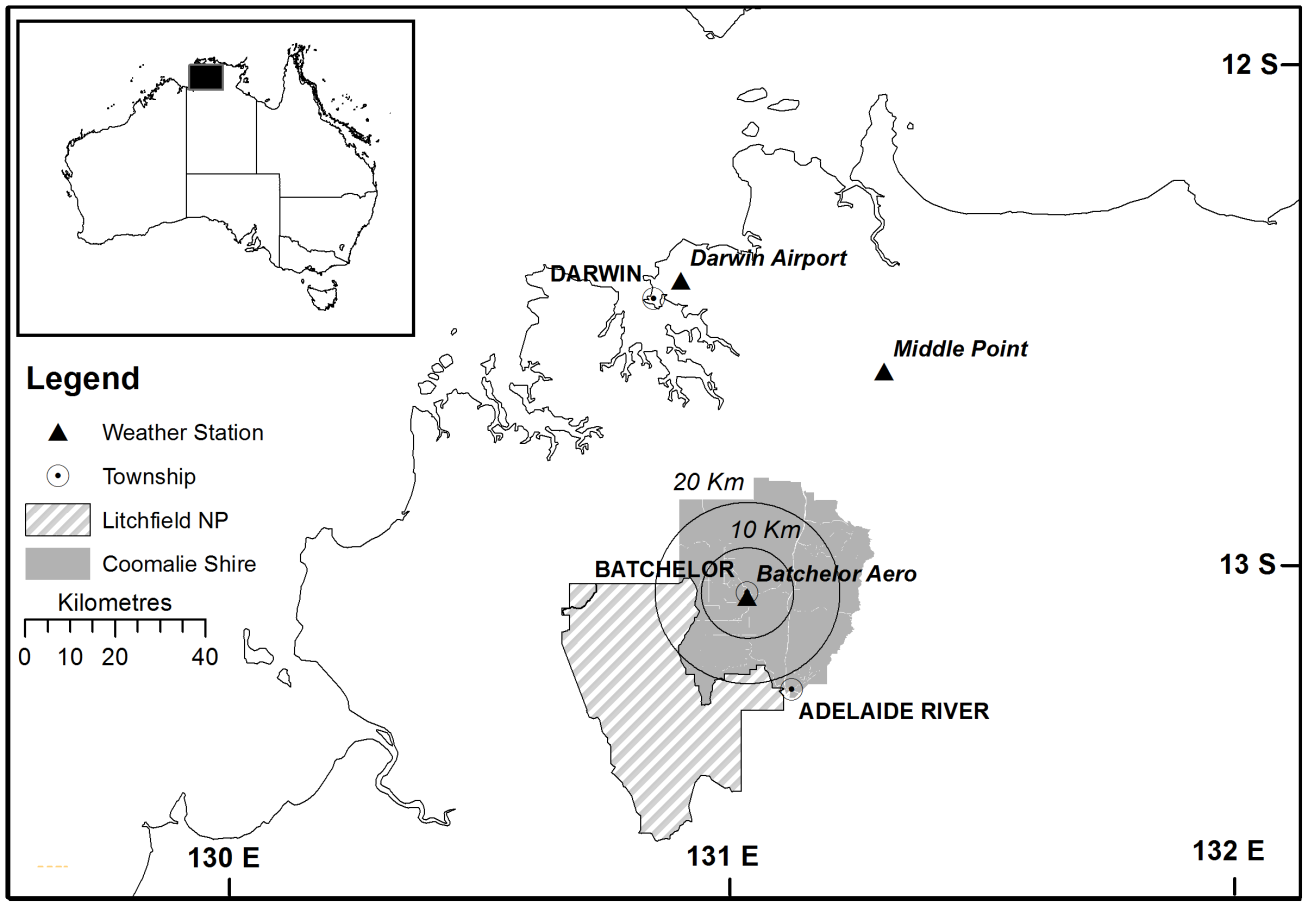
Location map showing: (1) study area (Coomalie Shire and Litchfield National Park) and (2) the Bureau of Meteorology weather stations (Batchelor Aero, Middle Point and Darwin Airport) in the Northern Territory, Australia. Circles showing 10 and 20 km radius around Batchelor were the areas used to determine fuel load.

The study area (∼1500 km^2^) included Coomalie Shire and northern area of Litchfield National Park, located approximately 70–100 km south of Darwin, Northern Territory (NT), Australia (Fig. 1). The region has a distinct wet-dry tropical climate. Air temperature is high throughout the year (mean maximum 33°C), while rainfall is highly seasonal (1662 mm, Batchelor Airport, Bureau of Meteorology, http://www.bom.gov.au) and concentrated in the wet season (November–April). The major vegetation type is savanna woodland dominated by *Eucalyptus miniata* (Cunn. Ex Schauer) and *E. tetrodonta* (F. Muell), with a grass understorey dominated by native perennial species such as *Heteropogon contortus* (L.) Roem. & Schult and *Alloteropsis semialata* (R. Br.) Hitchc. or annual grasses, such as *Sorghum intrans* (F.Muell. ex Benth.). The Coomalie Shire is a large rural region with a low human population (∼1,300), of which a significant proportion (28%) are indigenous [24]. It includes the townships of Bachelor and Adelaide River (Fig. 1). The majority of the Coomalie Shire is under private ownership for pastoral lease or semi-rural development [25], with other significant areas owned by local Aboriginal communities (the Finniss River Aboriginal Land Trust) or under Government ownership, including the Litchfield National Park [24].

The Coomalie region is the core area of dense invasion by the ‘Kent’ cultivar of the perennial C4 tussock grass *Andropogon gayanus* [18]. This cultivar ‘Kent’ was released in 1978 [15], planted in paddocks within the study area in the mid-1980s and spread was noticed from these paddocks to adjacent areas in the 1990s [26]. *A. gayanus* grows to 4 m tall and is physiologically active for longer into the dry season than the native grasses [18]. Consequently, in the early dry season (April/May), when much of the native herbaceous vegetation has senesced, *A. gayanus* puts on most of its growth, remains green and is clearly visible in the landscape. The intensity of *A. gayanus*-fuelled fires in the early dry season are greater than that reported for any native grass fires in northern Australia, even those lit in the late fire season [16].

### Determining the effect of *A. gayanus* invasion on regional fuel load

To determine the extent of *A. gayanus* cover at a regional scale, we undertook transect-based aerial surveys which are described in detail in Petty et al. (2012) [26]. In brief, helicopter surveys were undertaken between April and June 2009, when *A. gayanus* is most visually obvious in the landscape. Cover was estimated on a five-point scale (no *A. gayanus*, <1% (very low), 1–10% (low), 11–50% (medium), >50% (high)) following the Australian Government's national guidelines for mapping weeds [27]. A total of 109 helicopter-surveyed quadrats (175×250 m) were ground-truthed in September 2009. The accuracy of the survey was high (weighted Cohen's Kappa = 0.66; a completely random association has a score = 0 while complete agreement has a score = 1) [28]–[30]), with most of the error coming from individual or isolated clumps of plants that were undetected in the aerial survey [26]. The total area falling within each cover category was determined within two zones, one with the radius of 10 km and the other with a radius of 20 km around the township of Batchelor (13°0387′S; 131°0721′E), the centre of the main infestation area (Fig. 1).

The landscape fuel load (measured as tonne per hectare) within the study area was calculated as the product of the area within invasion density classes and the fuel load of each invasive density class. Data on *A. gayanus* and native grass fuel loads have been collected extensively within the study area since 2000 and reported as part of other studies (e.g. [14], [16], [18]). Fuel load was quantified as the dry weight of all fine fuel (<6 mm diameter grasses and non-photosynthetic woody material) harvested within 2 m^2^ quadrats (minimum 3 reps/site). The fine fuel load for native vegetation (i.e., the 0% *A. gayanus* cover category) used to calculate GFDI by the Bureau of Meteorology is set at 6 t ha^−1^ [31], based on the maximum native grass fuel loads measured by the CSIRO for these savannas (reported as 2–6 t ha^−1^) [32]. Although similar fuel loads have been reported (6.3 t ha^−1^, [33]), 6 t ha^−1^ is considered a high mean maximum fine fuel load for native grass savannas in the Top End region, and typically the result of fuel accumulation in the absence of fire for one or more years [34], [35]. We therefore applied the same criteria to determine fuel load of the other four categories of *A. gayanus* cover. For example, mean maximum fuel load for 100% *A. gayanus* cover in the absence of fire for one or more years in this region is 25.2 t ha^−1^ [16]. The fuel load for each cover category was calculated as the proportion of area in native cover and proportion in gamba cover, e.g. 5% *A. gayanus* cover equals 7 t ha^−1^ ( = 5% cover at 25.5 t ha^−1^ and 95% cover at 6 t ha^−1^). The midpoint of the fuel load ranges was used in the calculation of regional fuel load, so in this case, 5% cover represents the mid-point of the 1–10% cover category.

### Determining effect of increased regional fuel load on GFDI

To determine the effect of increases in regional fuel load resulting from invasion by *A. gayanus*, we used Purton's (1982) [22] modification of GFDI, which is defined as:
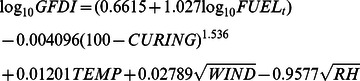
where *FUEL*_t_ is fuel load (t ha^−1^);

*CURING* is degree of curing (0–100%);

*TEMP* is air temperature (degrees Celsius);

*WIND* is wind speed (km h^−1^ at 10 m height in the open); and

*RH* is relative humidity (%)

Calculations were made for each day of two fire seasons (1 May to 31 October, 2008 and 2009) with *FUEL*_t_ equal to 6 t ha^−1^ and then with increasing 1 t ha^−1^ increments, up to 15 t ha^−1^. Calculations were made using hourly *TEMP*, *WIND* and *RH* data from three Bureau of Meteorology weather stations in the region (Batchelor Aero, 13.05°S, 131.03°E; Middle Point, 12.61°S, 131.30°E and Darwin Airport, 12.42°S, 130.89°E; Fig. 1). Daily *CURING* data was provided by Bushfires NT (the NT Government's fire authority) for the study period. This was provided daily to take into account annual and seasonal variations in fuel type and curing rate. *CURING* varied from 60% in the early dry season to 80–90% in the mid-dry season which was representative of data previously collected on the curing pattern of gamba grass (approximately 60% cured based on oven dry-weight measurements in the early dry-season, decreasing to 10–20% in the mid-late dry season; [14], [16]. Native grasses have been documented to be 19% and 11% cured in the early and late dry-season respectively based on oven dry-weight measurements [14]. The number of days/fire season in each of the six fire danger rating classes (low- catastrophic; Table 1) [36] was determined. The number of days on which GFDI>50 was determined for each weather station, as a function of fuel load. This is an important measure of change because GFDI>50 at *any* of the three weather stations triggers a fire weather warning/fire ban for the entire northern region of the NT. Therefore GFDI results from the three weather stations were assessed, and the total number of days GFDI>50 for the region was determined. ANOVA (Statistica 9.0 software package (StatSoft Inc, USA) was used to determine if the fuel load increase measured in this region resulted in a significant difference in the number of days with GFDI>50 (ANOVA factors Year (Fixed), Fuel load (Fixed).

**Table 1:** Australia's national forecast fire danger rating scale.

Grassland Fire Danger Index	Fire Danger Rating
0–11	Low/Moderate
12–24	High
25–49	Very high
50–74	Severe
75–99	Extreme
100+	Catastrophic

(Australian Emergency Management Committee (AEMC) 2009). A fire ban must be declared when GFDI>50.

### Quantifying the effect of increased fuel load on fire management costs

Once a fire ban is declared (GFDI>50, Table 1), Government fire management agencies must ensure that additional fire fighting equipment and staff resources are put on standby, and are available for call-out in the event of a wildfire. The cost of equipment required on stand-by was determined from records provided by Bushfires NT (NT Government) for 2007, 2008 and 2010. To enable comparison, data were converted to 2010 dollar values using the December quarter of nationwide Australian consumer price index (CPI). The economic cost of increasing stand-by resource costs under scenarios of increasing *FUEL*_t_ were determined by multiplying the 2010 cost of stand-by resources by the number of days with GFDI>50, calculated using *FUEL*_t_ at 6 t ha^−1^ and incremental increases up to 15 t ha^−1^.

To determine impacts of *A. gayanus* invasion on costs of wildfire control, we collated Bushfires NT reports on responses to individual wildfires over the past decade. Reporting prior to 2007 was less rigorous than current reporting, primarily because the resources required at that stage were relatively low. Where available, data were collected on the characteristics of fires (area covered, proportion of area invaded) and the site (e.g. cover of *A. gayanus*, assets within proximity of fire), and the resources used to control fire. Data were also collected on the total cost of fire management for the region, described by Bushfires NT as the Vernon Fire Control Zone. Data were adjusted to 2010 dollar values using nationwide Australian consumer price index using the December quarter.

## Results

### *A. gayanus* and the regional fuel load

*A. gayanus* invasion was extremely high at the landscape scale within the study area, with the largest area of medium and high invasion classes occurring close to the Batchelor township. Within 10 km radius of Batchelor, 18% of the aerially-surveyed quadrats were recorded with no *A. gayanus* cover and 44% of the quadrats were recorded with medium or high cover (Table 2). By comparison, in the 20 km radius around Batchelor, 50% of the plots were recorded with no *A. gayanus* and 21% of the quadrats were recorded with medium or high cover (Table 2). The estimated landscape fuel load had increased from the standard 6 t ha^−1^ (native grass fuel load), to approximately 10 t ha^−1^ and 8 t ha^−1^ respectively within 10 km and 20 km radius of Batchelor (Table 2).

**Table 2:** The cover and equivalent fuel load of *A. gayanus* in the surveyed in the Coomalie Shire, NT.

*A. gayanus* cover		Fuel load	Area (km^2^)		% Area	
Invasion Class	Cover (%)	t ha^−1^	10 km radius	20 km radius	10 km radius	20 km radius
Zero	0	6	22.9	267.8	18	50
Very Low	<1	6	19.1	74.6	15	14
Low	1–10	7	29.3	80.6	24	15
Medium	11–50	12	35.8	74.3	29	14
High	>50	20.5	17.3	36.2	14	7

### Effect of increased regional fuel load on GFDI

**Figure 2:**
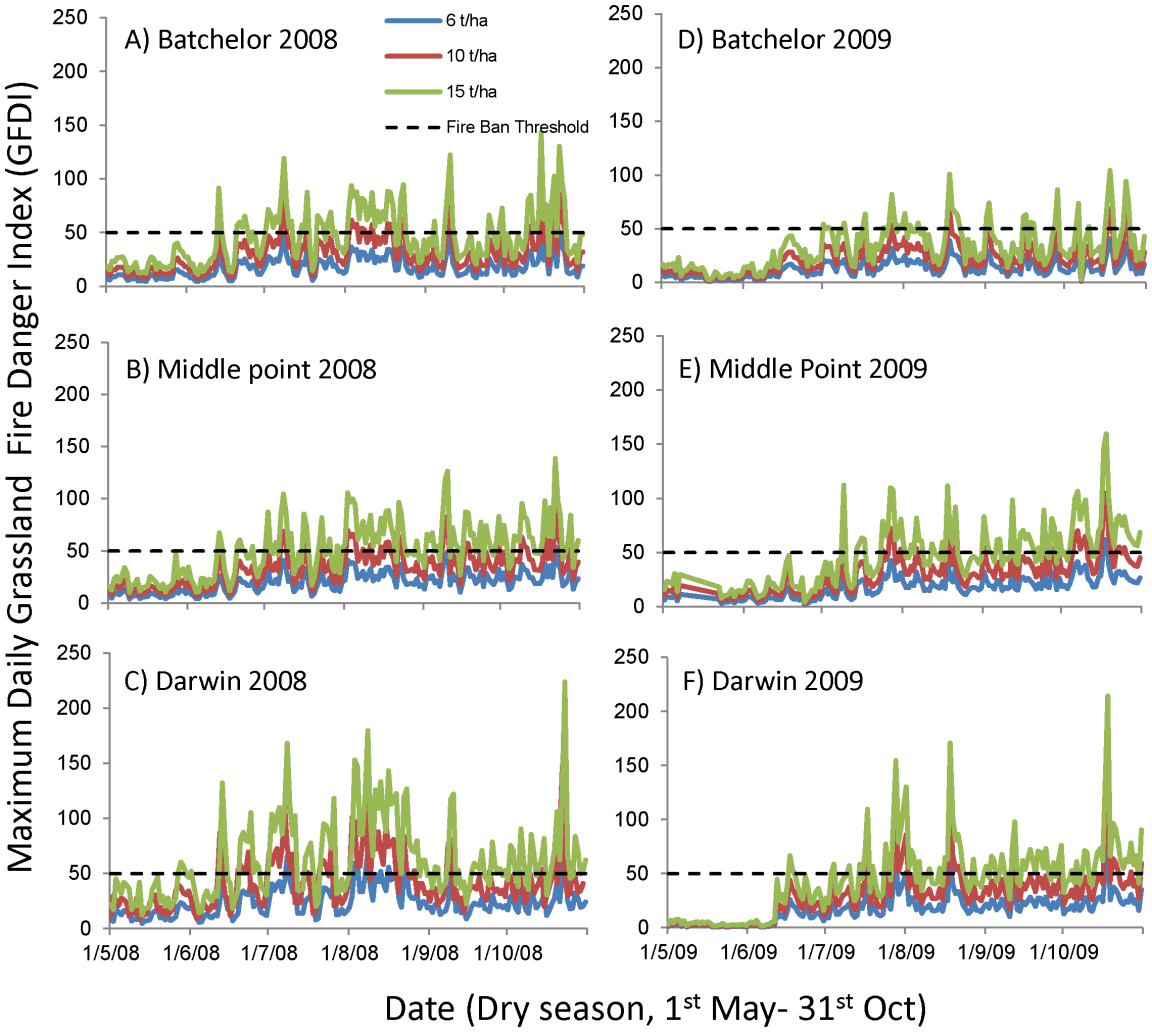
Daily maximum GFDI at three weather stations ((A, D) Batchelor, (B,E)Middle Point and (C,F) Darwin; Bureau of Meterology data) in 2008 and 2009 using three fuel load (*FUEL*_t_) scenarios: 6 (blue line), 10 (red line) and 15 t ha^−1^ (black line). GFDI of 50 is represented by the black line and is considered severe fire weather at which fire management authorities must declare fire ban days (Table 1).

**Figure 3:**
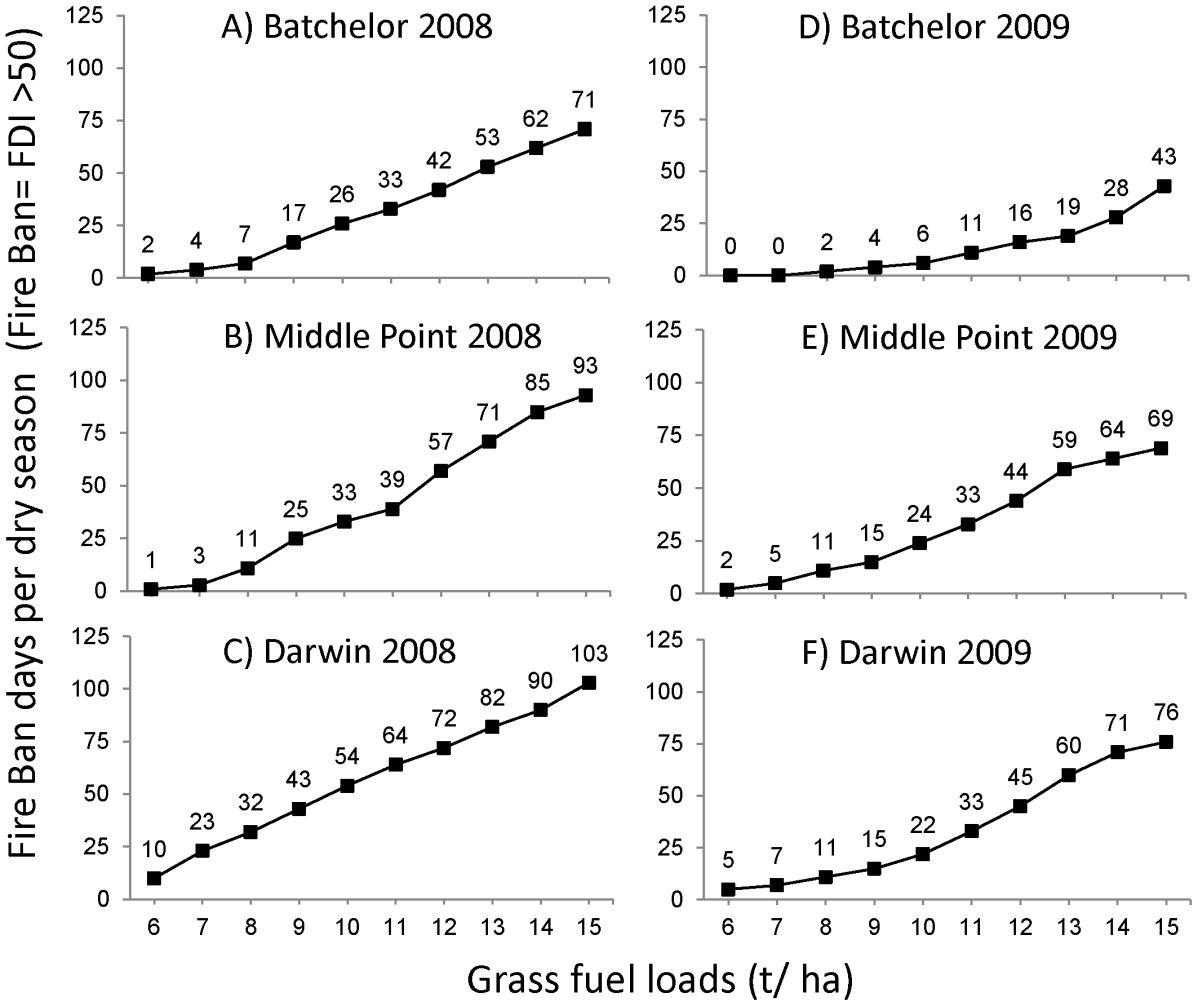
Number of days in the 2008 and 2009 fire season (1^st^ May to 31^st^ October) where the GFDI is ≥50, for the Batchelor, Middle Point and Darwin weather stations, NT, Australia. GFDI results are based on actual meteorological data from the three weather stations, fuel curing data and three different fuel load (*FUEL*_t_) scenarios (6, 10 and 15 t ha^−1^).

**Figure 4:**
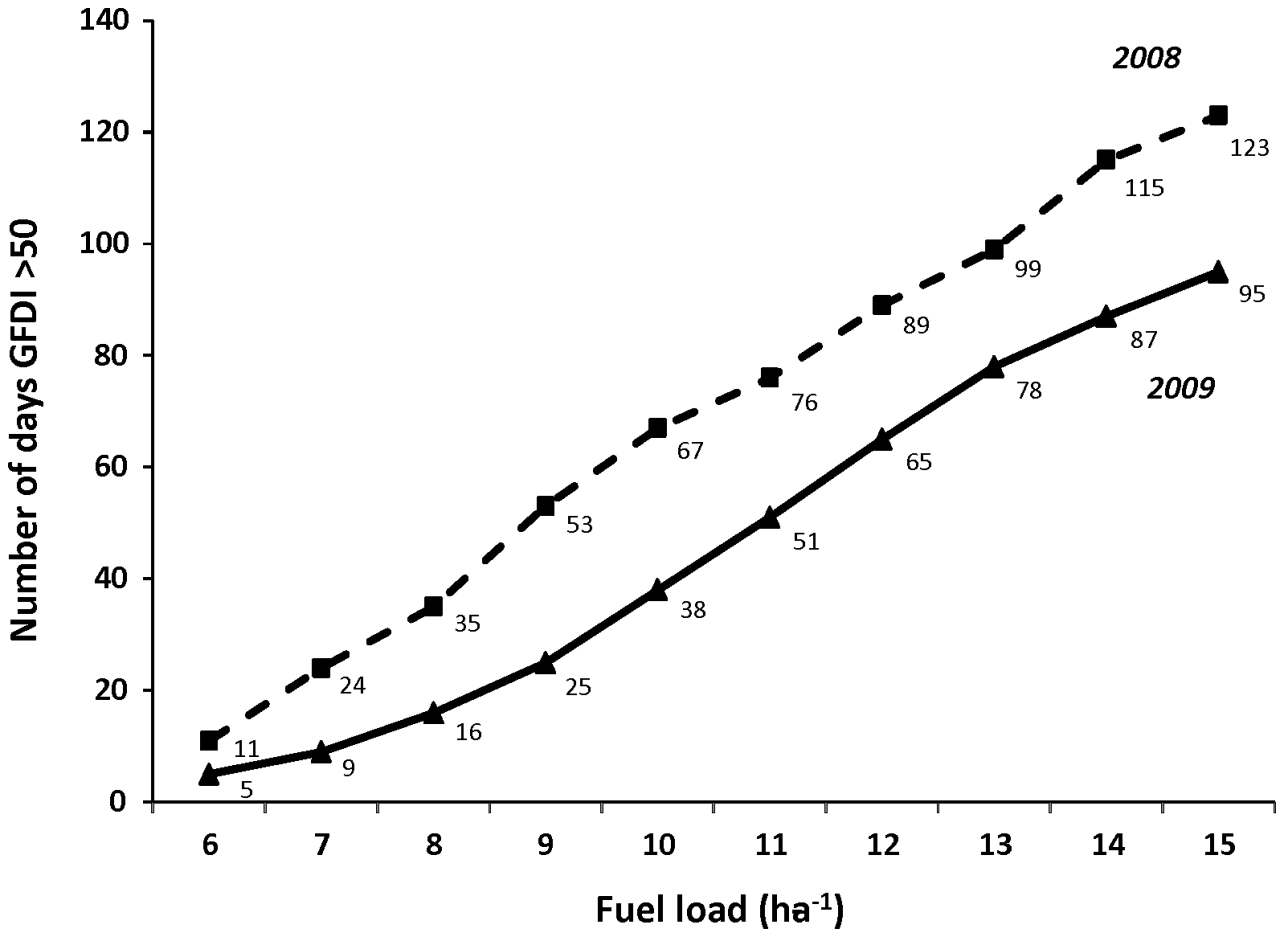
Number of fire ban days in the 2008 (dashed line) and 2009 (solid line) fire season for the Coomalie region, as a function of fuel load. A fire ban is declared when the GFDI≥50 at *any* of the three weather stations in the Coomalie region (Batchelor, Middle Point and Darwin).

The nature of the fire season changes if *FUEL*_t_ increases substantially (Fig. 2). Where *FUEL*_t_ = 6 t ha^−1^, the GFDI at Batchelor, Middle Point and Darwin weather stations remained in the low-moderate category (GFDI<12) for a substantial proportion of the fire season (Fig. 2) and GFDI reached ‘extreme’ (GFDI 75–99) at one weather station (Darwin) in both years of analysis. GFDI did not reach the ’catastrophic’ category (GFDI 100+) when *FUEL*_t_ = 6 t ha^−1^ (Fig. 2). The number of days with GFDI>50 was significantly higher in 2008 than 2009 (F_1,12_ = 5.2, P<0.05; Figs. 3 and 4). In both years, an increase in *FUEL*_t_ from 6 t ha^−1^ (native grass fuel) to 8 t ha^−1^ (the estimated fuel load in the 20 km radius around Bachelor) did not result in a significant change in number of days with GFDI>50, although this increased significantly when *FUEL*_t_ = 10 t ha^−1^, that is, the estimated fuel load in the 10 km radius around Bachelor (F_2,12_ = 9.8, P<0.01; Tukeys 6 = 8<10 t ha^−1^). When *FUEL*_t_ = 6 t ha^−1^, the number of days with GFDI>50 at individual weather stations varied from zero (Batchelor, 2009) to ten days (Darwin, 2008), whereas when *FUEL*_t_ = 10 t ha^−1^, the number of days with GFDI>50 at individual weather stations ranged between 6 days (Batchelor, 2008) to 54 days (Darwin, 2008) (Fig. 2, Fig. 3). The variation between weather stations resulted from their location (Fig. 1), with Darwin closest to the coast and reporting higher wind speeds. The number of days in the Coomalie region with GFDI>50 increased markedly with each tonne of fuel (Fig. 4). Modeling based on the 2008 weather data resulted in 11 days with GFDI>50 when *FUEL*_t_ = 6 t ha^−1^ which increased to 67 and 123 days when *FUEL*_t_ = 10 and 15 t ha^−1^, respectively. Similarly using 2009 weather data, there were 5 days with GFDI>50 when *FUEL*_t_ = 6 t ha^−1^, which increased to 38 and 95 days when *FUEL*_t_ = 10 and 15 t ha^−1^, respectively. This means that at fuel loads of 15 t ha^−1^, 67% of days in 2008 fire season, and 52% of days in the 2009 fire season, would exceed GFDI 50 and therefore would be declared total fire ban days. The duration of the severe fire season extended considerably (Fig. 2), with the first day with GFDI>50 occurring on 28th July in 2009 when *FUEL*_t_ = 6 t ha^−1^, whereas it occurs 6 weeks earlier (17^th^ June) when *FUEL*_t_ = 10 t ha^−1^. A similar pattern occurred using 2008 weather data. The fire season ends with wet season rains and this is not affected by fuel load.

### Effect of increased fuel load on fire management costs

The cost per day of equipment that were required to be ‘on stand-by’ in readiness for fighting wildfires had increased by 30 times from 2007 to 2010 ($375 to $11,442; Table 3, NT Government, unpublished data). Prior to 2007, the resources on stand-by were two staff members and a 4-Wheel Drive fitted with fire-fighting equipment. For a 4-week period in 2008, Bushfires NT trialed the use of fire-fighting aircraft, including a fixed-winged water-bombing aircraft with a 3000 L water holding capacity, and a rotary wing helicopter fitted with 500 L capacity helicopter fire bucket (the ‘bambi bucket’; SEI Industries Ltd, Delta, British Columbia, Canada). Due to the success of these tools in managing the high intensity *A. gayanus* grass fires, the Bushfires NT altered their policies and by 2010, required that a fixed-wing aircraft and two water-bombing helicopters were available for use on fire ban days. In addition, three staff were required, including at least one skilled in coordinating aerial fire-fighting campaigns. Therefore, the cost of stand-by resources in 2010 was $11,442/day (2010 dollars NT Government, unpublished data). Clearly, this has substantial implications for the cost of fire management. For example, this equates to an additional $640,000 annually using the 2008 weather data, and $378,000 using the 2009 weather data when *FUEL*_t_ was increased from 6 t ha^−1^ to 10 t ha^−1^ when calculating GFDI (Table 4; NT Government, unpublished data).

**Table 3:** Cost of fire management stand-by equipment in 2007, 2008 and 2010.

Equipment	Rate/day	2007	2008	2010
Plane	$3,572	$0	$3,572	$3,572
Helicopter	$2,210	$0	$2,210	$4,420
Water truck	$1,100	$0	$1,100	$1,100
Loader	$ 800	$0	$ 800	$1,600
Grass Fire Unit	$ 375	$ 375	$ 750	$ 750
*Total cost*		**$375**	**$ 8,432**	**$11,442**

Data are provided in 2010 dollars and include GST.

**Table 4:** Estimated cost of stand-by equipment ($11,442 per day; Table 3) per year, based on the number of days GFDI>50 in three different *FUEL*_t_ scenarios (6, 10 or 15 t ha^−1^) at three weather stations (Fig. 3).

Estimated cost of stand-by equipment for fire ban days (per year)			
	Grass Fuel Loads		
	6 t ha^−1^	10 t ha^−1^	15 t ha^−1^
2008	$125,862	$766,614	$1,407,366
2009	$57,210	$434,796	$1,086,990

Data in 2010 dollars. Costs do not include staffing costs.

The *total* cost of fire management in the region (Vernon Fire Control zone) was nine times higher in 2010–2011 compared to the cost prior to invasion in 2002–2003 ($1,335,000 *c.f.* $185,000; Table 5; NT Government, unpublished data). This increase was largely driven by the increase in wildfire response costs, which was $860,000 in 2010–2011 compared to $224,000 in 2006–7 (2010 dollars; NT Government, unpublished data). The increased total cost reflected the substantial increase in staff and resources allocated to individual wildfire events. The comparison of pre- and post-invasion records of wildfire control at six sites in the study area showed a significant increase in the average cost per fire event, from $938 (±$252) pre-invasion to $25,609 (±$5134) post-invasion (Table 6; Table S1; NT Government, unpublished data). Marked increases in cost were primarily the consequence of the introduction and on-going use of the water bombing aircraft described above. The water bombing aeroplane and helicopters cost at approximately $3,570 hr^−1^ and $2100 hr^−1^ respectively. The aircraft were deployed in five of the six fires documented in *A. gayanus* areas, with between 12 and 21 hours of use (NT Government, unpublished data).

**Table 5:** Fire management costs in the Vernon Fire Control region (2002–2011).

	VERNON		
	Operational & mitigation costs	Wildfire control costs	Total costs
	$,000	$,000	$,000
2010–11	475	860	1,335
2009–10	341	499	840
2008–09	273	684	957
2007–08	135	543	678
2006–07	258	224	482
2005–06	N/A	N/A	310
2004–05	N/A	N/A	214
2003–04	N/A	N/A	184
2002–03	N/A	N/A	185

Data were adjusted to 2010 dollar values based on nationwide Australia CPI, using December quarter for adjustment. Costs do not include GST. Operational and mitigation costs include the costs of Bushfires NT staff salaries and resources to prevent and mitigate the damage caused by late season wildfires. Wildfire costs are the costs of responding to and extinguishing a wildfire.

**Table 6:** Comparison of costs (in 2010 dollar values) from six paired fires in (a) native grass, (prior to *A. gayanus* grass invasion) and (b) *A. gayanus* fuelled fires.

Fire Location	Cost	
	(a) Native grass	(b) *A. gayanus*
Rum jungle	$750	$20,171 (70% cover)
Tortilla	$375	$23,687 (70% cover)
Batchelor	$375	$6,194 (70% cover)
Batchelor mine	$750	$32,672 (90% cover)
Darwin River	$1,500	$27,209 (80% cover)
Lake Bennett	$1,875	$43,723 (35% cover)

Paired fires were selected based on the closeness of site of ignition, therefore the staff and resource response would be expected to be similar over time. For a full breakdown of fire costs see Table S1 in Supporting Information. Data are provided in 2010 dollars.

## Discussion

The impact of invasive species on fuel properties has been described in many ecosystems globally [5]. Fire is a principal ecological driver of the structure and function of the savanna ecosystems, therefore concerns have been repeatedly raised about the potentially dramatic impacts of high biomass non-native grasses [14], [37]–[39]. *Andropogon gayanus* invasion is now widespread in the Northern Territory's *Eucalyptus*-dominated tropical savanna, and this study demonstrates substantial regional-scale effects on fuel load and costs of mitigating fire risk.

### *A. gayanus* invasion has increased regional fire loads

The rapid rate of spread of *A. gayanus* and its ability to invade the broad range of savanna habitats [26], [37] suggests that this species could result in major fire and weed management issues across a vast region of northern Australia. The Kent cultivar of *A. gayanus* was released relatively recently, having been promoted in the 1980's as an improved pasture species. Yet, by 2008, the area of invasion outside pastoral systems covered 15,000 km^2^ of the NT alone, with large areas invaded in Western Australia and Queensland [15]. The core site of invasion was mapped in this study. In the 10 km radius zone around the Batchelor township, over 20.5 km^2^ now has high *A. gayanus* cover (>50%), and this increases to 36 km^2^ in the extended 20 km radius zone (Table 2). Of particular concern is the additional 75 km^2^ of surveyed land in the very low cover category (<1%), because the transition from individual clump to medium and high cover occurs quickly (2 to 5 years), particularly in more suitable habitats such as riparian corridors [26], [37]. The extensive area of low and very low invasion is therefore very likely to have higher cover classes within the next decade, resulting in significantly more challenging and more expensive fire management programs.

This study clearly demonstrates that increased fuel load causes increased fire risk, as measured by GFDI. Given the importance of this index to inform fire managers and the public about fire danger [40], it is critical that the parameters used to calculate GFDI are representative of conditions in the region. An ongoing evaluation of fuel load over the broad area of invasion is required to improve the accuracy of risk calculations. This will include updating estimates as both the area invaded, and the cover within invaded region, increases. Remote sensing and LIDAR technologies can provide more spatially comprehensive data than methods used in this study [41], [42], but only at significant cost. However, such data would support the cost-effective deployment of resources required to manage fire risk every year in this region. In addition, changes in patterns of curing and their influence on GFDI should also be evaluated.

### *A. gayanus* invasion substantially increased fire management costs

Fire officers within the study area began altering their approach to wildfire control in invaded areas in 2005 to mitigate the increased risk they were experiencing from individual fire events. For example, they increased the number of fire officers attending fires, and increased their personal safety by upgrading to full “structural” personal protection clothing that is rated as suitable for responding to structure & forest fires. They are the only grass-fuel fire-fighting group in Australia to adopt this heavier and more flame-retardant uniform. The changes progressed to use of a water-bombing helicopter in 2008 (Northern Territory Government 2008) and access to multiple fire-fighting aircraft, on-ground equipment such as earthmovers and water-tankers, and increased numbers of fire-fighting staff in 2010. These changes are reflected in the region's total fire management budget. Between 2002 and 2005, the total cost of fire management remained relatively stable (between $185,000–$214,000 in 2010 dollars). At that time, limited records of the costs of controlling individual fire events were kept. However, this changed in 2006–7 financial year, when there was a substantial increase in total fire management costs (∼$482,000) in the Coomalie region, and the cost of wildfire control alone was $224,000 (all figures in 2010 dollars). Due to a further increase in wildfire management costs in 2007–8 (to $678,000), the NT Government provided Bushfires NT with a once-off increase in funding to their operating budget to increase their capacity to undertake more fire prevention burning in the early dry season and to purchase additional fire-fighting equipment, including a >$160,000 front end loader [43]. The focus on fire prevention meant that wildfire management costs remained relatively stable until 2010–2011, when costs jumped to a total of approximately $1.3 million, of which approximately $860,000 was for wildfire control. The operating costs of aircraft for aerial fire-fighting are a major component of these costs. The use of both rotary (∼$1200/hr) and fixed-wing aircraft (∼$1700/hr) are a direct response to changed fire regimes, and can only be used immediately within two ‘primary response zones’ in the Batchelor region. Bushfires NT has defined the Primary Response Zones as those areas in the Batchelor region that are subject to special Fire Ban and Fire Warning requirements, due to the density of high biomass non-native grass species [44]. Due to operating costs, equipment are not to be deployed outside these dense invasion areas without significant justification [44].

The increase in ‘operational and wildfire mitigation’ costs for the study area reflect the significant increase in the cost of enacting stand-by procedures that now include aerial fire-fighting and earthmoving equipment and increased number of staff. In 2010, as a consequence of the regional assessment of fuel load in this study, Bushfires NT and Bureau of Meteorology trialled the use of calculating GFDI with *FUEL*_t_ = 9 t ha^−1^ for two weather data stations (Darwin and Middle Point) and *FUEL*_t_ = 11 t ha^−1^ for Batchelor weather station. This resulted in 28 days in the 2008 fire season with a GFDI>50, and 9 days in the 2009 fire season. Differences between years were due to weather conditions, particularly the number of days with high wind speed in 2008. Fourteen days occurred without a significant fire event, i.e. stand-by equipment were not deployed, at a cost of $150,000 for Bushfires NT, and an additional fourteen days when aerial and other fire-fighting equipment was deployed at a cost of approximately $330,000. The costs of the latter were reported in the wildfire management component of the budget.

### Synthesis and application

This study expands our understanding of the impact of non-native invasive plants by demonstrating the economic impact of a high biomass invader on fire risk mitigation. There would be many cases in which land managers have faced increased costs due to plant invasions changing fire regimes in many ecosystems [45], [46], yet we could not find examples where they have been documented and used to modify weed management programs. Understanding the economic consequences of plant invasions is necessary to consider the appropriate funding level and approaches to strategically manage invading species in order to limit further increases in impact costs [47]. In this study, increased expenditure on fighting fires fuelled by *A. gayanus* invasion was funded by the government's fire authority and, therefore, not considered within *A. gayanus* management planning by local weed management agencies. This disconnect leads to obvious problems, both to the level and type of resources directed to the issue. In the case study area, funding for *A. gayanus* control is substantially lower than the costs of fire management, and does not address the goal of mitigating risk across a broader region. The data suggest that if the current scenario continues, the fire hazard from *A. gayanus* will represent a major environmental and economic problem in the next decade.

This situation demonstrates the consequences of not responding to the early warnings of weed risk with appropriate management. The need for management was clear even in the information submitted for registration of the cultivar [48] which described unintended “spread downwind” at pastoral trial sites. Despite government pastoral researchers noting as early as 1990 that “the need for proper management cannot be overlooked with this species which restricts it to smaller more controlled areas” [49], the cultivar was planted widely, as a pastoral grass and for minesite rehabilitation, including by broadscale aerial sowing. Obvious spread and impacts were documented during the 1990's and 2000's [18], [50], [51]. However, the species was not declared a noxious weed until 2008 by which time deliberate plantings and subsequent invasion were widespread in northern Australia.

In summary, this research demonstrates the importance of improving our knowledge of major impacts of invasive species and ensuring that this knowledge is used to revise and improve management plans. Quantification of ecological and economic impacts is both informative and powerful for changing policy. This case study has already changed policies on fire management, and will be critical in scenario planning with stakeholders to ensure that strategic decisions about weed management are made.

## Supporting Information

Table S1 **Comparison of fire costs.** Comparison of costs (in 2010 dollar values) from six paired fires in (a) native grass (prior to *A. gayanus* grass invasion) and (b) *A. gayanus* fuelled fires. For (b), the cover of A. gayanus in the burnt area was provided by fire fighters and reported for each. Paired fires were selected based on the close proximity of ignition therefore the staff and resource response would be expected to be similar over time. Grass Fire Unit is standard 4WD with basic fire fighting equipment. Enhanced vehicle has additional communications and fire-fighting capabilities. Private Vehicle is the rate paid for call out of staff private vehicle. Helicopter, grader, loader and water tanker rates vary slightly depending on the contractor available for the fire. Two different helicopters used during this fire, giving a different rate per hour.
(DOCX)
