# Supplementary material for: Adding Fuel to the Fire: The Impacts of Non-Native Grass Invasion on Fire Management at a Regional Scale
Source: PLoS One. 2013 May 14;8(5):e59144. doi: 10.1371/journal.pone.0059144 (PMC3653883; doi:10.1371/journal.pone.0059144)
Supplement: Table S1 — Comparison of fire costs. Comparison of costs (in 2010 dollar values) from six paired fires in (a) native grass (prior to A. gayanus grass invasion) and (b) A. gayanus fuelled fires. For (b), the cover of A. gayanus in the burnt area was provided by fire fighters and reported for each. Paired fires were selected based on the close proximity of ignition therefore the staff and resource response would be expected to be similar over time. Grass Fire Unit is standard 4WD with basic fire fighting equipment. Enhanced vehicle has additional communications and fire-fighting capabilities. Private Vehicle is the rate paid for call out of staff private vehicle. Helicopter, grader, loader and water tanker rates vary slightly depending on the contractor available for the fire. Two different helicopters used during this fire, giving a different rate per hour. (DOCX) [file pone.0059144.s001.docx]

| **Table S1.** Comparison of fire costs  Comparison of costs (in 2010 dollar values) from six paired fires in (a) native grass (prior to *A. gayanus* grass invasion) and (b) *A. gayanus* fuelled fires. For (b), the cover of A. gayanus in the burnt area was provided by fire fighters and reported for each. Paired fires were selected based on the close proximity of ignition therefore the staff and resource response would be expected to be similar over time. Grass Fire Unit is standard 4WD with basic fire fighting equipment. Enhanced vehicle has additional communications and fire-fighting capabilities. Private Vehicle is the rate paid for call out of staff private vehicle. Helicopter, grader, loader and water tanker rates vary slightly depending on the contractor available for the fire. Two different helicopters used during this fire, giving a different rate per hour | | | | | | | | | | | | | | | |
| --- | --- | --- | --- | --- | --- | --- | --- | --- | --- | --- | --- | --- | --- | --- | --- |
| **Paired Fires 1: *Rum Jungle*** | |  | | | (a) Native grass | | | | | (b) *A. gayanus* (70% cover) | | | | | |
|  | | **Rate/unit** | | | **Day or Hours** | | **Total** | | | **Day or Hours** | | | **Total** | | |
| Backhoe | | $110/hr | | | - | | - | | | 6 hr | | | $660 | | |
| Water-bombing aircraft | | $1078/hr | | | - | | - | | | 4.5 hr | | | $4,851 | | |
| Water-bombing helicopter | | $974/hr | | | - | | - | | | 9.5 hr | | | $9,250 | | |
| Water Tanker | | $110/hr | | | - | | - | | | 6 hr | | | $660 | | |
| Grass Fire unit | | $375/day | | | 2 days | | $750 | | | 6 days | | | $2,250 | | |
| Enhanced Vehicle | | $500/day | | |  | |  | | | 5 days | | | $2,500 | | |
|  | |  | | | ***Total*** | | $750 | | | ***Total*** | | | $20, 171 | | |
|  | | | | | | | | | | | | | | | |
| **Paired Fires 2: *Tortilla*** | | |  | | | (a) Native grass | | | | | | (b) *A. gayanus* (70% cover) | | | |
|  | | | **Rate/unit** | | | **Day or Hours** | | | **Total** | | | **Day or Hours** | | **Total** | |
| Grader | | | $138/hr | | | - | | | - | | | 10 hr | | $1,386 | |
| Water-bombing aircraft | | | $1675/hr | | | - | | | - | | | 2.55 hr | | $4,272 | |
| Water-bombing helicopter | | | $1195/hr | | | - | | | - | | | 9.5 hr | | $11,354 | |
| Water Tanker | | | $110/hr | | | - | | | - | | | 10 hr | | $1,100 | |
| Grass Fire Vehicle | | | $375/day | | | 1 day | | | $375 | | | 5 days | | $1,875 | |
| Enhanced vehicle | | | $500/day | | |  | | |  | | | 7 days | | $3,500 | |
| Private Vehicle | | | $200/day | | | - | | | - | | | 10 hr | | $200 | |
|  | | |  | | | ***Total*** | | | $375 | | | ***Total*** | | $23,687 | |
|  | | | | | | | | | | | | | | | |
| **Paired Fires 3: *Batchelor*** |  | | | (a) Native grass | | | | | | | (b) *A. gayanus* (70% cover) | | | | |
|  | **Rate/unit** | | | **Day or Hours** | | | | **Total** | | | **Day or Hours** | | | | **Total** |
| Loader | $143/hr | | | - | | | | - | | | 14 hr | | | | $2,002 |
| Grass Fire Unit | $375/day | | | 1 day | | | | $375 | | | 5 days | | | | $1,875 |
| Private Vehicle | $200/day | | | - | | | | - | | | 1 day | | | | $200 |
| Enhanced Vehicle | $500/day | | | - | | | | - | | | 4 days | | | | $2,000 |
|  |  | | | ***Total*** | | | | $375 | | | ***Total*** | | | | $6,077 |

| **Paired Fires 4: *Batchelor Mine*** |  | (a) Native grass | | (b) *A. gayanus* (80% cover) | |
| --- | --- | --- | --- | --- | --- |
|  | **Rate/unit** | **Day or Hours** | **Total** | **Day or Hours** | **Total** |
| Water-bombing aircraft | $1675/hr | - | - | 5.23 hr | $8,762 |
| Water-bombing helicopter | $1232/hr | - | - | 16.2 hr | $19,952 |
| Water tanker | $110/hr | - | - | 2 hr | $220 |
| Grass Fire Unit | $375/day | 2 days | $750 | 7 | $2,625 |
| Enhanced vehicle | $500/day |  |  | 2 days | $1000 |
|  |  | ***Total*** | $750 | ***Total*** | $32,559 |

| **Paired Fires 5: *Darwin River*** |  | (a) Native grass fire | | (b) *A. gayanus* (80% cover) | |
| --- | --- | --- | --- | --- | --- |
|  | **Rate/unit** | **Day or Hours** | **Total** | **Day or Hours** | **Total** |
| Loader | $192/day | - | - | 21 hr | $4,043 |
| Water-bombing aircraft | $1675/hr | - | - | 4 hr | $6,702 |
| Water-bombing helicopter | $1204/hr | - | - | 7.7 hr | $9,269 |
| Water tanker | $110/hr | - | - | 2 hr | $220 |
| Grass Fire Unit | $375/day | 4 days | $1,500 | 13 days | $4,875 |
| Enhanced Vehicle | $500/day | - | - | 3 days | $1,500 |
| Private Vehicle | $200/day | - | - | 3 days | $600 |
|  |  | ***Total*** | $1,500 | ***Total*** | $27,209 |

| **Paired Fires 6: *Lake Bennett*** |  | (a) Native grass fire | | (b) *A. gayanus* (35% cover) | |
| --- | --- | --- | --- | --- | --- |
|  | **Rate/unit** | **Day or Hours** | **Total** | **Day or Hours** | **Total** |
| Grader | $185/day | - | - | 17.5 hr | $3245 |
| Loader | $176/day | - | - | 35 hr | $6160 |
| Water-bombing aircraft | $1675/hr | - | - | 5.67 hr | $9,499 |
| Water-bombing helicopter | $931/hr* | - | - | 16.25 hr | $15,136 |
| Water tanker | $121/hr | - | - | 10 hr | $1,210 |
| Grass Fire Unit | $375/day | 5 days | $1,875 | 6 days | $2250 |
| Enhanced Vehicle | $500/day | - | - | 9 days | $4,500 |
| Private Vehicle | $200/day | - | - | 3 days | $600 |
|  |  | ***Total*** | $1,875 | ***Total*** | $42,600 |
